# Mice with induced pulmonary morbidities display severe lung inflammation and mortality following exposure to SARS-CoV-2

**DOI:** 10.1172/jci.insight.145916

**Published:** 2021-06-22

**Authors:** Reut Falach, Liat Bar-On, Shlomi Lazar, Tamar Kadar, Ohad Mazor, Moshe Aftalion, David Gur, Yentl Evgy, Ohad Shifman, Tamar Aminov, Ofir Israeli, Inbar Cohen-Gihon, Galia Zaide, Hila Gutman, Yaron Vagima, Efi Makdasi, Dana Stein, Ronit Rosenfeld, Ron Alcalay, Eran Zahavy, Haim Levy, Itai Glinert, Amir Ben-Shmuel, Tomer Israely, Sharon Melamed, Boaz Politi, Hagit Achdout, Shmuel Yitzhaki, Chanoch Kronman, Tamar Sabo

**Affiliations:** 1 Department of Biochemistry and Molecular Genetics,; 2 Department of Pharmacology, and; 3 Department of Infectious Diseases, Israel Institute for Biological Research, Ness-Ziona, Israel.

**Keywords:** COVID-19, Mouse models

## Abstract

Mice are normally unaffected by SARS coronavirus 2 (SARS-CoV-2) infection since the virus does not bind effectively to the murine version of the angiotensin-converting enzyme 2 (ACE2) receptor molecule. Here, we report that induced mild pulmonary morbidities rendered SARS-CoV-2–refractive CD-1 mice susceptible to this virus. Specifically, SARS-CoV-2 infection after application of low doses of the acute lung injury stimulants bleomycin or ricin caused severe disease in CD-1 mice, manifested by sustained body weight loss and mortality rates greater than 50%. Further studies revealed markedly higher levels of viral RNA in the lungs, heart, and serum of low-dose ricin–pretreated mice compared with non-pretreated mice. Furthermore, lung extracts prepared 2–3 days after viral infection contained subgenomic mRNA and virus particles capable of replication only when derived from the pretreated mice. The deleterious effects of SARS-CoV-2 infection were effectively alleviated by passive transfer of polyclonal or monoclonal antibodies generated against the SARS-CoV-2 receptor binding domain (RBD). Thus, viral cell entry in the sensitized mice seems to depend on viral RBD binding, albeit by a mechanism other than the canonical ACE2-mediated uptake route. This unique mode of viral entry, observed over a mildly injured tissue background, may contribute to the exacerbation of coronavirus disease 2019 (COVID-19) pathologies in patients with preexisting morbidities.

## Introduction

Severe manifestations of coronavirus disease 2019 (COVID-19) are mostly restricted to distinct groups of people: elderly persons and individuals who have preexisting morbidities form a significantly high proportion of people who develop acute lung injury and acute respiratory distress syndrome (ALI/ARDS) and therefore require intensive care (1, 2). These observations suggest that the development of severe COVID-19 requires an underlying pathological condition or predisposition in addition to the actual SARS coronavirus 2 (SARS-CoV-2) infection. In the laboratory, most COVID-19 animal models based on ferrets, hamsters, and nonhuman primates develop a mild pathology that resolves within a relatively short period of time (3–5), reflecting the more prevalent asymptomatic-to-mild manifestation of the disease observed in humans. Mice, on the other hand, seem to be totally refractive to SARS-CoV-2 infection because of the inability of the virus to bind effectively to the murine version of the angiotensin-converting enzyme 2 (ACE2) receptor molecule (6). To overcome this hurdle, mice animal models in which human ACE2 is transiently or constitutively expressed have been established (6–8). Although severe clinical manifestations were observed in some of these genetically modified mouse models, these were not dependent on preexisting morbidities and may have been due to high-level, indiscriminate expression of human ACE2 in a wide spectrum of cells that do not normally express this molecule (8).

Bacterial LPS and bleomycin are well-established stimulants of ALI/ARDS in animal models (9). Ricin, a plant-derived toxin, has also been proven in recent years to cause ALI/ARDS (10, 11). In the present study, we examined whether induction of a relatively mild and transient lung injury in genetically unaltered CD-1 mice by application of carefully chosen sublethal doses of these stimulants would promote susceptibility to SARS-CoV-2. Indeed, after infection with SARS-CoV-2 via i.n. application, mice pretreated with low-dose ricin (LDR) or bleomycin displayed a significant and sustained decrease in body weight, and a significant proportion of these mice died within a surveillance period of 2 weeks. Passive transfer of polyclonal or monoclonal antibodies directed against SARS-CoV-2 or the receptor binding domain (RBD) portion of the viral spike resulted in full protection of LDR mice after viral infection. Our findings imply a noncanonical mode by which SARS-CoV-2 invades cells in the presence of a preexisting tissue injury. A mechanism similar to this yet unresolved route of viral internalization may contribute to the aggravation of COVID-19 manifestations in patients with preexisting morbidities. Finally, the animal model presented in the current study is important for its ability to support unaltered SARS-CoV-2 replication in genetically nonmodified mice.

## Results

### Induced pulmonary injuries predispose mice to SARS-CoV-2 infection.

**Figure 1:**
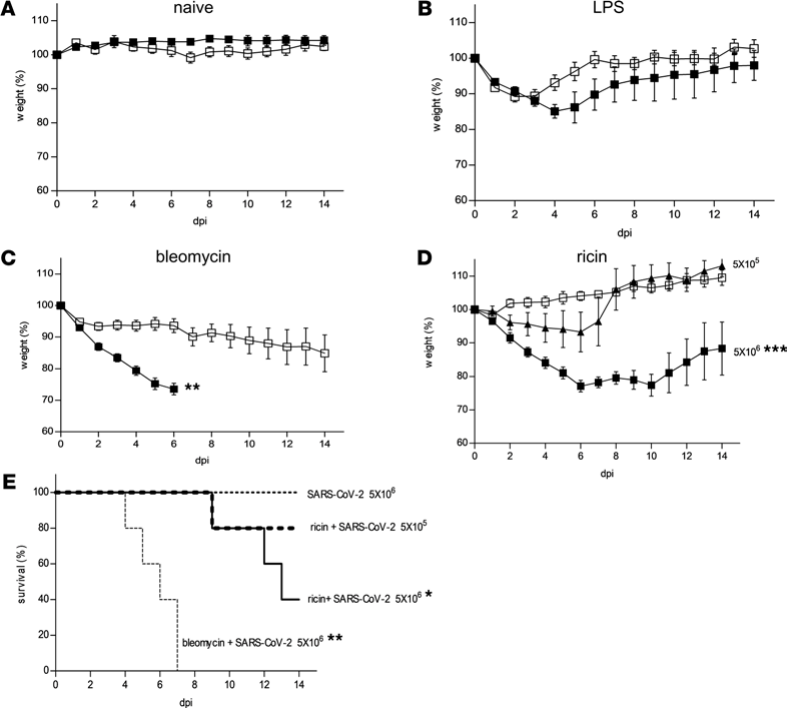
Effects of SARS-CoV-2 infection on body weight and mortality of CD-1 mice pretreated with ALI/ARDS stimulants. (**A**–**D**) Body weights of CD-1 mice (presented as percentage of original weight determined at the time of viral instillation) were monitored over a period of 15 days after i.n. instillation of SARS-CoV-2. Data are mean ± SEM, *n* = 5–6 per group, analyzed using 2-way ANOVA followed by Bonferroni’s posttests, *P < 0.05, **P < 0.01, ***P < 0.001 compared with no virus. Displayed representative experiment out of 3–5 independent experiments for each treatment. (**A**) Body weights of mice infected with virus at a dose of 5 × 10^6^ PFU per mouse (black squares) compared with body weights of naive mice (white squares). (**B**) Mice were administered LPS (1.7 mg/ kg body weight) and 1 day later were infected (black squares) or not (white squares) with virus (5 × 10^6^ PFU/mouse). (**C**) Mice were administered bleomycin (2 U/kg body weight) and 4 days later were infected (black squares) or not (white squares) with virus (5 × 10^6^ PFU/mouse). Because of significant mortality, only data of 0 to 6 days are presented for bleomycin–SARS-CoV-2 mice. (**D**) Mice were administered ricin (1.7 μg/ kg body weight) and 2 days later were infected with virus at a dose of 5 × 10^5^ (black triangles) or 5 × 10^6^ (black squares) PFU/mouse or not (white squares). (**E**) Kaplan-Meier survival curves of the mouse groups exhibiting mortality: dotted line is SARS-CoV-2 (5 × 10^6^ PFU/mouse), thick dashed line is LDR–SARS-CoV-2 (5 × 10^5^ PFU/mouse), black line is LDR–SARS-CoV-2 (5 × 10^6^ PFU/mouse), thin dashed line is bleomycin–SARS-CoV-2 (5 × 10^6^ PFU/mouse). Data were analyzed using log-rank (Mantel-Cox) test. *n* = 5.

Cell entry of SARS-CoV-2 requires the binding of the viral spike to ACE2. Unlike ACE2 of humans, hamsters, and ferrets, ACE2 of mice does not sensitize cells for infection (12). In line with this fact, CD-1 mice were refractive to SARS-CoV-2 infection, as demonstrated by the lack of any discernable reduction in their body weights after viral infection (Figure 1A). To determine whether induction of pulmonary comorbidities affect the susceptibility of mice to SARS-CoV-2, CD-1 mice were treated with 1 of 3 selected stimulants of ALI: LPS, bleomycin, or ricin. To promote the development of a mild and transient injury that resolves within a few days, these stimulants were i.n. administered at low doses, 1.7 mg/kg, 2 U/kg, and 1.7 μg/kg of LPS, bleomycin, and ricin, respectively. The time point at which the mice displayed an onset of disease as exemplified by body weight loss was 1, 4, and 2 days after the administration of LPS, bleomycin, or ricin, respectively. At these time points, the preadministered mice were infected with SARS-CoV-2 at a dose of 5 × 10^6^ PFU/mouse and then monitored for 15 days. LPS-pretreated mice displayed an additional decrease in body weight after viral infection; however, body weight regain began at 5 days after infection and the mice approached their weights determined prior to viral infection within a short period of time (Figure 1B). In contrast, mice pretreated with either bleomycin or ricin displayed a more sustained decline in body weight, which lasted throughout the entire surveillance period after infection with SARS-CoV-2, reaching as low as 70% (bleomycin, Figure 1C) and 77% (ricin, Figure 1D) of their original weights determined immediately prior to viral infection. Most notably, 100% and 50% of the bleomycin- and ricin-pretreated mice, respectively, died within the 15-day surveillance period: death occurred at 4 to 7 days after infection for bleomycin-pretreated mice and 9 to 13 days after infection for ricin-pretreated mice (Figure 1E).

The present study focused mostly on the SARS-CoV-2 mouse model predisposed by LDR. Repeated experiments allowed us to determine that after infection with SARS-CoV-2 at a dose of 5 × 10^6^ PFU per mouse, death rates were within the range of 17%–75% with an overall average of 39% (*n* = 26, 5 independent experiments). When LDR mice were subjected to SARS-CoV-2 infection at a lower viral dose, 5 × 10^5^ PFU per mouse, body weight reduction was less pronounced (<7%, Figure 1D) and full weight regain was reached at 8 days after infection. In a representative experiment (Figure 1E), 20% of the animals died, similar to the average death rate determined in repeated experiments (*n* = 16, 3 independent experiments, range = 0%–40%). Taken together, these findings clearly demonstrated that although CD-1 mice were refractive to SARS-CoV-2 infection, compromised pulmonary conditions induced by preexposure to low doses of selected ALI stimulants conferred a SARS-CoV-2–sensitive phenotype to these mice.

### Characterization of the pulmonary injury induced by i.n. application of LDR.

**Figure 2:**
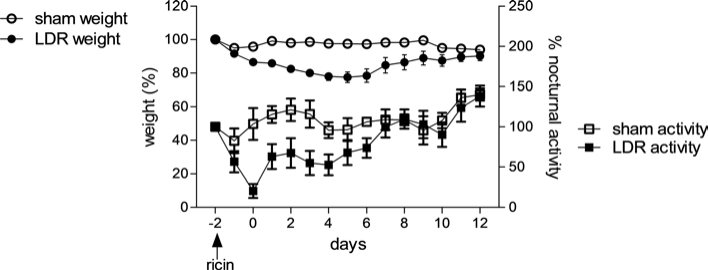
Effects of LDR application on body weight and motor activity of CD-1 mice. CD-1 mice were monitored over a period of 15 days after i.n. application (indicated by arrow) of ricin (1.7 μg/kg) or PBS (sham treatment): circles, body weight of LDR (black) and sham (white) mice, squares: nocturnal motor activity profiles of LDR (black) and sham (white) mice. Body weights are expressed as a percentage of original weight determined at the time of ricin or sham administration. Data represent mean ± SEM, *n* = 6; nocturnal activity is expressed as percentage of activity determined prior to treatment. Data represent mean ± SEM of cage activity, *n* = 4 cages, 6 mice per cage.

To characterize the impaired pulmonary background that promotes SARS-CoV-2 sensitivity, we monitored the pathological changes occurring in LDR mice. These examinations, which were carried out starting from the time of ricin application, included surveillance of body weight and motor activity over time, differential blood counts, and bronchoalveolar fluid (BALF) analyses. In repeated experiments, LDR mice that were not subjected to viral infection displayed a transient loss of weight, and maximal weight reduction occurred 2–6 days after administration of LDR; commencement of body weight regain was recorded between 3 and 7 days after ricin treatment (Figure 2). In most experiments, mice reached their initial body weights at days 7–12, and average body weight percentage at day 12 was 102.5% ± 2.5%. LDR mouse morbidity was also monitored by following their motor activity, utilizing a recently developed computerized home cage monitoring system (HCMS100) based on laser-beam interruption counts (13). Examination of nocturnal activity profiles showed that LDR mice displayed a transient reduction in motor activity compared with sham-treated mice (*P* < 0.001 at days 0–3), which resolved at day 7 (Figure 2).

Hematological analysis was performed on blood samples collected from LDR mice 2 days after the LDR was administered (day 0); this time point corresponded to that at which LDR mice were infected with the virus. White blood cell counts in general and neutrophils and monocytes in particular were elevated. Platelet counts increased as well (Supplemental Table 1; supplemental material available online with this article; https://doi.org/10.1172/jci.insight.145916DS1). BALF collected from LDR mice at this time point displayed higher contents of total protein and cholinesterase, a serum-resident enzyme whose aberrant presence in the lungs attests to impairment of the lung/blood barrier. Cell counts and TNF-α, IL-1β, and IL-6 levels were also elevated in the BALF of the LDR mice compared with sham-treated mice (Supplemental Table 1). In line with this observation, indicating that an inflammatory reaction has launched in response to LDR administration, gene expression of cytokines and chemokines in the lungs of LDR mice 2 days after ricin application was significantly upregulated in comparison with naive mouse lungs (Supplemental Figure 1). Finally, assessment of ricin catalytic activity (28S rRNA depurination) in the lungs demonstrated that approximately 8% of lung 28S rRNA extracted from LDR mice 2 days after administration was depurinated at this time point (Supplemental Table 1). At day 7, nearly all of the hematological and BALF-related parameters displayed values that did not differ significantly from the corresponding values determined in sham-treated mice (a single exception was the BALF total protein concentration value, which was significantly higher than in the sham mice). In addition, depurinated 28S rRNA, the hallmark of ricin catalytic activity, could not be measured above background level at day 7 (Supplemental Table 1). Taken together, these observations suggest that the impaired pulmonary state in LDR mice was limited, with resolution occurring within a matter of days.

To provide a more comprehensive view of the alterations in protein expression that might play a role in sensitizing LDR mice to SARS-CoV-2 infection, whole lung cell RNA-Seq of LDR-treated versus naive mice was performed (Supplemental Figure 2). Principal component analysis revealed distinct transcriptional signatures between naive and LDR mice (Supplemental Figure 2A). Compared with the naive group, there were 8545 differentially expressed genes (DEGs) in the LDR group (2 days after LDR application), comprising 4394 upregulated and 4151 downregulated genes (Supplemental Figure 2B). Gene Ontology (GO) analysis ranked by the FDR (*q* value) revealed 1379 GO terms in upregulated DEGs (biological process: 865, cellular function: 210, molecular function: 304) and 1289 GO terms in downregulated DEGs (biological process: 810, cellular function: 160, molecular function: 319) after eliminating redundant terms. These results reflect the notable magnitude of alterations that occurred in the transcriptome profile of the LDR mice in a wide range of genes and in all 3 domains of the gene annotation analysis. Observing the top 50 (displaying highest *q* values, see Supplemental Table 2) upregulated biological processes GO terms showed an overrepresentation of themes related to immune and inflammatory response (44%) (Supplemental Figure 2C), corroborating our observation that LDR application stimulates expression of proinflammatory gene pathways, even though it does not cause a clinically acute disease.

**Figure 3:**
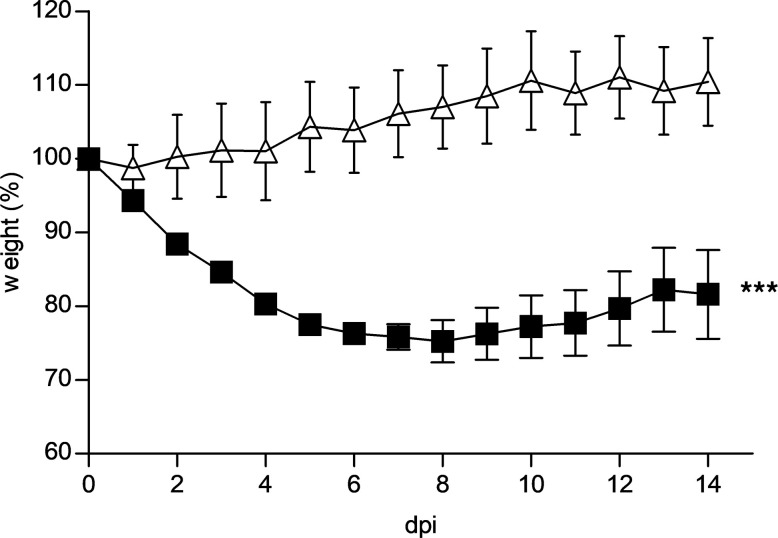
Anti-ricin treatment does not alleviate SARS-CoV-2–induced body weight loss of LDR mice. Two days after i.n. application of LDR (1.7 μg/kg), mice were treated (i.p.) with anti-ricin antibodies (1730 IsNU/mouse; ref. 14) and 2 hours later were infected (black squares) or not (white triangles) with SARS-CoV-2 (5 × 10^6^ PFU/mouse). Body weights of CD-1 mice were monitored over a period of 15 days after viral infection. Data, presented as percentage of original weight determined at the time of virus instillation, are mean ± SEM, *n* = 4–5 per group, analyzed using 2-way ANOVA followed by Bonferroni’s posttests, ***P < 0.001 compared with no virus. A representative experiment out of 3 independent experiments is displayed.

We further ascertained that the marked deterioration observed in LDR–SARS-CoV-2 mice was directly due to the viral infection and did not reflect an exacerbation of a ricin-related disease. To this end, LDR–SARS-CoV-2 mice were treated 2 hours prior to SARS-CoV-2 infection with equine-derived anti-ricin F(ab′)_2_ antibodies at a more than 10-fold higher dose than that required for full protection of mice from a lethal dose (7 μg/kg body weight) of ricin (14, 15). Indeed, treatment of mice with these antibodies at the time of LDR treatment prevented body weight loss (data not shown), attesting to efficient and rapid neutralization of the toxin. Nevertheless, treatment with anti-ricin antibodies did not have any measurable beneficent effect on the well-being of the LDR–SARS-CoV-2 mice; after viral infection, weight continued to decline markedly (Figure 3) in a manner similar to that observed in LDR–SARS-CoV-2 mice that were not treated with anti-ricin antibodies (Figure 1D). Within the 15-day surveillance period, 20% of the LDR–SARS-CoV-2 mice treated with anti-ricin antibody died; this death rate fell within the death rate range documented for mice that were not subjected to anti-ricin antibody treatment. Thus, the significant signs of morbidity documented in LDR mice infected with SARS-CoV-2 stemmed from the viral infection and not from the toxic activity of continuous ricin.

### Characterization of the SARS-CoV-2 infection model in LDR mice.

**Figure 4:**
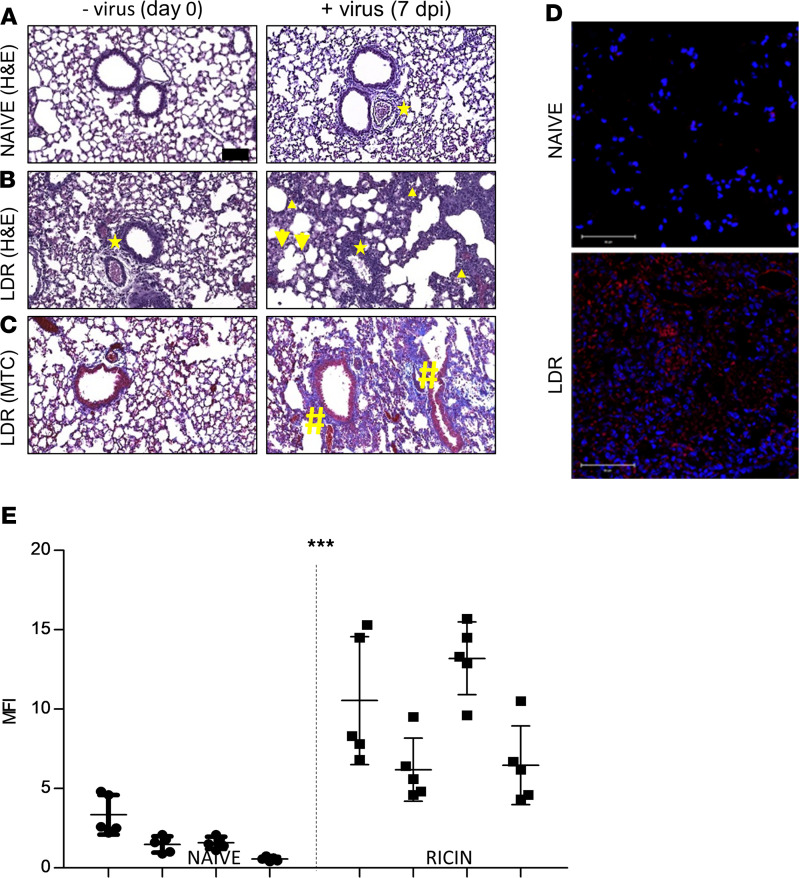
SARS-CoV-2 binding and injury in lungs of LDR mice. (**A**–**C**) Histology: naive (**A**) and LDR (**B** and **C**) mice (ricin application at day –2) were subjected to SARS-CoV-2 infection at a dose of 5 × 10^6^ PFU/mouse. Lungs at the time of viral infection (day 0) and 7 days later were harvested, fixed, and processed for paraffin embedding. Sections (5 μm) were stained with H&E for general histopathology (**A** and **B**) or with Masson’s trichrome (MTC) for collagen (**C**). Representative sections of *n* = 4 mice are shown. Indicated are perivascular and peribronchial inflammation (stars), infiltration in perivascular and alveolar sites (triangles), edema (arrows), and fibrin (hashtags). Magnification ×20, scale bar: 100 μm. (**D** and **E**) Confocal microscopy scans of lung sections. (**D**) Sections from LDR mice (lungs harvested 2 days after LDR administration) and naive mice were incubated with SARS-CoV-2 (1000 PFU/mL), immunostained with a mAb directed against the spike region of SARS-CoV-2, and then visualized by AF594-labeled anti-human antibody. Staining of SARS-CoV-2 in red and identification of nuclei by DAPI in blue, scale bar: 50 μm (see also Supplemental Figure 3). Representative sections of *n* = 4 mice are shown. (**E**) Scatterplots of the fluorescence staining intensities of SARS-CoV-2 expressed as MFI ± SEM, *n* = 4 mice, 5 scanned fields/lung, analyzed using unpaired *t* test. ***P <0.001. Abbreviation: dpi, days after infection.

We evaluated the impact of SARS-CoV-2 infection on lung histopathology using H&E and Masson’s trichrome staining (Figure 4). At 7 days after infection, lungs from LDR–SARS-CoV-2 mice exhibited severe damage manifested by extensive peribronchial and perivascular inflammatory cell infiltration along with intra-alveolar edema and fibrin and macrophage accumulation (Figure 4, B and C, right). In contrast, lungs from SARS-CoV-2 mice (Figure 4A, right) exhibited a relatively preserved alveolar structure and mild immune peribronchial and perivascular cell infiltration. Upon viral infection (Figure 4, day 0), LDR mice exhibited mild mononuclear cell infiltrations in the peribronchial and perivascular areas (Figure 4, B and C, left). Taken together, these observations showed that severe manifestations of SARS-CoV-2–induced lung injury were restricted to LDR mice. To determine whether this observed increase in injury may be related to an increase in binding of SARS-CoV-2 to the lung tissue of LDR mice, we performed comparative immunohistochemical analyses to detect viral binding. To this end, lung sections prepared from LDR-treated and naive mice were incubated with SARS-CoV-2 and then immunostained with a mAb generated against the viral spike, after which viral binding was visualized by confocal microscopy (Figure 4, D and E, and Supplemental Figure 3). Indeed, lungs of LDR mice displayed markedly higher levels of SARS-CoV-2 binding than lungs of naive mice (MFI of viral binding to naive and LDR mice lung sections = 1.74 ± 0.27 and 9.09 ± 0.88, respectively, *P* < 0.0001), establishing that the ALI stimulant–induced lung injury increased SARS-CoV-2 tropism toward murine lung tissue.

**Figure 5:**
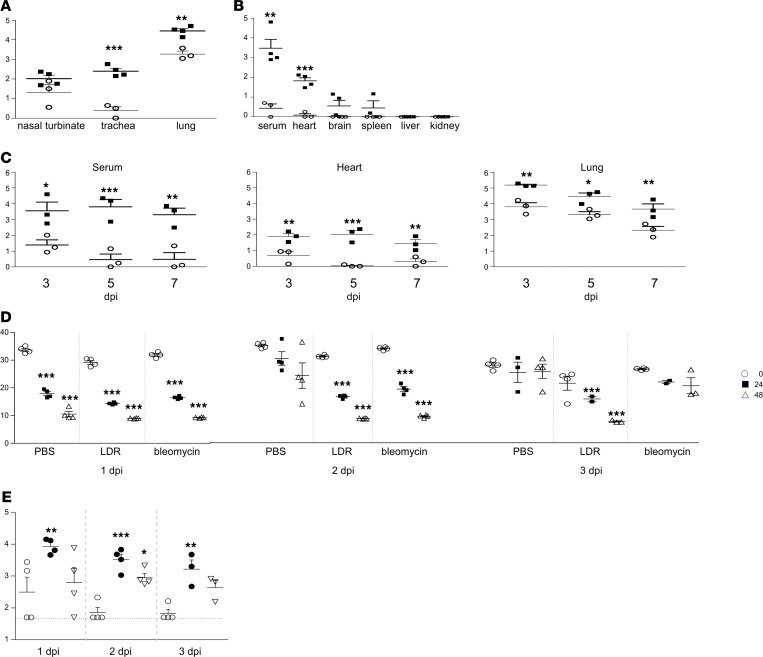
Viral RNA levels and viability. (**A**–**C**) Viral RNA levels in various tissues. RT-PCR was performed on tissue homogenates prepared from SARS-CoV-2 mice (empty circles) and LDR–SARS-CoV-2 mice (filled squares) at 2 days after infection (**A** and **B**) or at 3, 5, and 7 days after infection (**C**). A PFU calibration curve tested in parallel was utilized to express Ct values as calculated PFUs. (**D**) Viral growth kinetics. Lung homogenates prepared from SARS-CoV-2, LDR–SARS-CoV-2, and bleomycin–SARS-CoV-2 mice at 1–3 days after infection were monitored for the presence of viable virus in an in vitro Vero E6 cell culture–based growth kinetics assay (see Methods). Decreasing Ct values over time determined by RT-PCR of cell growth medium samples collected at 0, 24, and 48 hours indicate viral replication in the Vero cells. Asterisks represent statistical significance between adjacent time points of sampling (24 hours compared with *t* = 0, 48 hours compared with 24 hours) (**E**) Subgenomic mRNA analysis. Quantitative RT-PCR of E gene sgmRNA was performed on sham-treated (empty circles), LDR-treated (filled circles), and bleomycin-treated (empty triangles) mice lung homogenates prepared 1–3 days after infection with SARS-CoV-2 (5 × 10^6^ PFU/mouse) Asterisks represent statistical significance in comparison to sham-treated mice (empty circles). Data represent mean ± SEM, *n* = 3–4 per group, analyzed using 2-way ANOVA followed by Bonferroni’s posttests, *P < 0.05, **P < 0.01, ***P < 0.001.

To further characterize the effect of LDR administration on SARS-CoV-2 infectability, we exposed naive and LDR mice to SARS-CoV-2 at a dose of 5 × 10^6^ PFU per mouse, and thereafter various organs and tissues were harvested and examined for the presence of viral RNA. At 2 days after viral infection, viral RNA levels in nasal turbinate, trachea, and lung homogenates were markedly higher in LDR–SARS-CoV-2 mice than in SARS-CoV-2 mice (Figure 5A). Significantly higher levels of viral RNA were also documented as early as 2 days after infection in the serum and heart of LDR–SARS-CoV-2 mice (Figure 5B). Higher levels of viral RNA were also detected in brain and spleen homogenates of LDR–SARS-CoV-2 mice; however, these did not differ statistically from the corresponding levels determined in SARS-CoV-2 mice. No viral RNA was found in liver and kidney homogenates (Figure 5B). We further profiled viral RNA in serum and heart homogenates over time. Indeed, LDR–SARS-CoV-2 mice displayed significantly high levels of viral RNA in the serum and heart at all time points examined — 3, 5, and 7 days after infection (Figure 5C). Examination of lung homogenates at these time points demonstrated a progressive decline of viral RNA over time, yet levels remained relatively high even in SARS-CoV-2 mice (Figure 5C). To determine whether the viral RNA measured in the lungs could be related to intact viable virus, lung homogenate samples were subjected to growth kinetics profiling. To this end, Vero E6 cells were incubated with lung homogenate samples prepared at 1, 2, and 3 days after infection, and extracellular viral RNA was quantified by real-time PCR (RT-PCR) in growth medium samples removed at the start of incubation (*t* = 0) and then again after 24 and 48 hours. Unlike lung homogenates of SARS-CoV-2 mice (Figure 5D, PBS), RT-PCR of homogenates prepared at 2 and 3 days after infection from LDR–SARS-CoV-2 mice displayed decreasing Ct values over time (Figure 5D, LDR). This finding attests to the fact that the virus retained its viability at later time points solely in LDR–SARS-CoV-2 mice.

This experiment was further extended to investigate whether viable virus particles are also present in the lungs of bleomycin-pretreated mice (Figure 5D, bleomycin). Indeed, as in LDR–SARS-CoV-2 mice, virus particles capable of multiplying were also detected in the lungs of bleomycin–SARS-CoV-2 mice, but the viability phase in these mice was shorter than in LDR–SARS-CoV-2 mice; viable virus was detected in bleomycin–SARS-CoV-2 mice at 2 days after infection but not at 3 days after infection. Finally, to prove that the virus indeed undergoes multiplication in the lungs of mice that were pretreated with LDR or bleomycin, we inspected the mouse lung extracts for the presence of subgenomic mRNA (sgmRNA, Figure 5E). Significant levels of sgmRNA were detected in the lungs of ricin-pretreated and bleomycin-pretreated mice at 2 and 3 days after infection but not in the lungs of mice that were not subjected to pretreatment. In line with the data observed in the growth kinetics analysis, sgmRNA levels were more pronounced in the lungs of the LDR–SARS-CoV-2 mice. Taken together, these findings attest to viral multiplication specifically in the lungs of mice pretreated with ALI stimulant.

### Antiviral antibodies protect LDR mice from SARS-CoV-2 infection.

**Figure 6:**
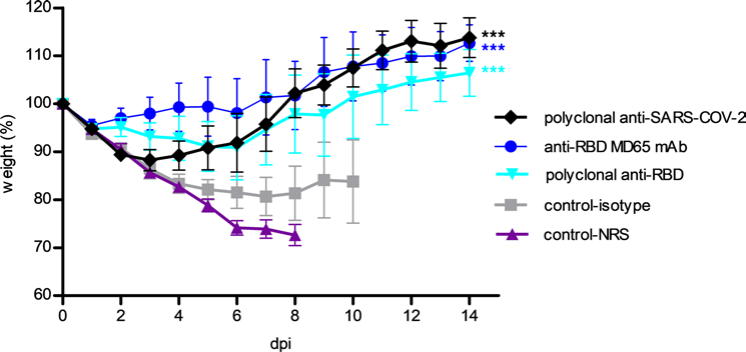
Anti–SARS-CoV-2–related antibodies protect LDR mice from SARS-CoV-2 infection. Two days after i.n. application of LDR (1.7 μg/kg), mice were treated (1 mL/mouse, i.p.) with the following SARS-CoV-2–related antibodies: rabbit-derived polyclonal anti–SARS-CoV-2 antibodies (black diamonds), rabbit-derived polyclonal anti-RBD antibodies (magenta triangles), and anti-RBD MD65 mAb (blue circles). Unrelated isotype control (gray squares) and normal rabbit serum (cyan, red triangles) served as negative controls for the SARS-CoV-2–specific monoclonal and polyclonal antibodies, respectively. Mouse body weights were monitored over a period of 15 days after viral infection. Because of significant mortality, only data of 0 to 8–10 days are presented for the control groups. Data, presented as percentage of original weight determined at the time of virus instillation, represent mean ± SEM, *n* = 4–5 per group, ***P < 0.001, analyzed using 2-way ANOVA followed by Bonferroni’s posttests, compared with their corresponding control antibodies. Displayed representative experiment out of 3 independent experiments.

If indeed the deleterious manifestations documented in LDR–SARS-CoV-2 mice were due to direct viral activity, one may expect them to be redressed by treating the mice with anti–SARS-CoV-2–related antibodies. To address this issue, LDR mice were treated with polyclonal antibodies raised against either the entire virus or the RBD portion of the SARS-CoV-2 spike (see Methods) or with the human MD65 mAb shown to target the SARS-CoV-2 RBD (13). Application of any of the 3 anti–SARS-CoV-2–related antibodies 2 hours prior to viral infection promoted mouse body weight regain (Figure 6). Accordingly, at 9–10 days after infection, the body weights of the group of mice treated with each of the 3 antibody preparations approached their weight values determined at the time of SARS-CoV-2 infection. No deaths were recorded in the groups of mice treated with any of the antiviral antibodies. In contrast, mice treated with nonspecific antibodies (normal rabbit serum or TL1 mAb isotype control; ref. 16) displayed a steady drop in body weight, and 40%–100% of the mice succumbed within 5–13 days after infection.

### Effect of LDR pretreatment on ACE2 and transmembrane protease, serine 2, expression.

**Figure 7:**
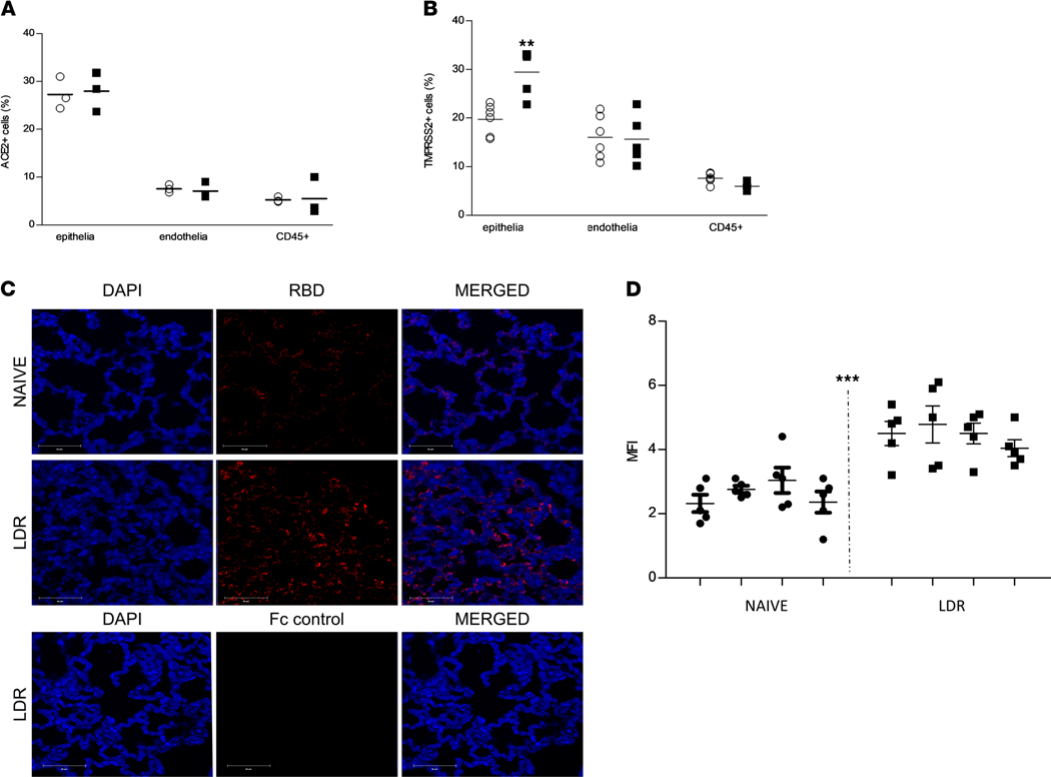
Expression of ACE2 and TMPRSS2 and RBD binding in lung cells of LDR mice. (**A** and **B**) Flow cytometry analysis of lung cells harvested from LDR (filled bars) and sham-treated (empty bars) mice 2 days after treatment. ACE2 (**A**, *n* = 3), TMPRSS2 (**B**, *n* = 6). Data referring to percentage of positive cells out of the indicated cell types are presented as mean ± SEM, analyzed using 2-way ANOVA followed by Bonferroni’s posttests, **P < 0.01. (**C** and **D**) Confocal microscopy scans of lung sections. (**C**) Sections from LDR (2 days after LDR administration) and naive mice were incubated with RBD-huFc or AChE-huFc (Fc control) and then immunostained with anti-human AF594. Staining of RBD in red and identification of nuclei by DAPI in blue. Scale bar: 50 μm. (**D**) Scatterplots of the fluorescence staining intensities of RBD expressed as MFI ± SEM. *n* = 4 mice, 5 scanned fields/lung, analyzed using unpaired *t* test, ***P < 0.001.

ACE2 is the key cell surface receptor for SARS-CoV-2, and the transmembrane protease, serine 2 (TMPRSS2), protease serves as a cofactor involved in the trimming of the viral spike, a process that facilitates viral internalization (17). It has been well established by others and shown in the present study that mice are not susceptible to SARS-CoV-2 infection, and as such they cannot serve as models for COVID-19. This refractivity toward the virus is attributed to the inability of SARS-CoV-2 to bind effectively to murine ACE2 (12). Yet, in view of the fact that low doses of selected ALI stimulants rendered mice sensitive to the virus, we examined whether application of these stimulants affected the expression of ACE2 and TMPRSS2. To this end, lungs of LDR mice were harvested 2 days after application of ricin. This time point was chosen because alterations documented at this time point correspond to those that occur at the time of viral infection after treatment with LDR. Cell suspensions prepared from mouse lungs were analyzed by flow cytometry utilizing anti–murine ACE2 (anti-mACE2) and anti-TMPRSS2 antibodies (Figure 7, A and B, and Supplemental Figure 4).

Comparison between sham-treated and LDR mice demonstrated that ricin administration did not bring about any noticeable increase in the number of cells, epithelia, endothelia, or CD45^+^ cells expressing ACE2 (Figure 7A). In contrast, the number of lung cells expressing TMPRSS2 was significantly higher in LDR mice than in the sham-treated mice (Figure 7B). Notably, this increase in TMPRSS2 expression was cell type dependent. Thus, a 50% increase was observed in the number of TMPRSS2^+^ lung cells of epithelial (CD45^–^CD31^–^) lineage, whereas lung endothelial (CD45^–^CD31^+^) and hematopoietic cell (CD45^+^) TMPRSS2^+^ counts (Figure 7B) were not affected.

The fact that the number of cells expressing cell-bound ACE2 did not increase after treatment with ricin does not negate the possibility that viral entry into lung cells of LDR mice takes place by the binding of SARS-CoV-2 spike RBD to the murine ACE2 with the aid of factor(s) that are present only after induction of the pulmonary injury or by binding to cell surface receptors other than ACE2 that are newly expressed/exposed after mouse sensitization. To address this issue, we examined whether application of LDR affects binding of RBD to mouse lung cells. To this end, lung sections prepared from LDR-treated and naive mice were incubated with RBD-huFc fused protein (18) or with AChE-huFc fused protein (used as control for Fc; ref. 19), then immunostained with AF594-labeled anti-huFc antibodies and visualized by confocal microscopy (Figure 7, C and D). Lungs of LDR mice displayed markedly higher binding of RBD-huFc than those of naive mice (Figure 7C). This staining reflected authentic RBD binding to the lung cells and not huFc binding, in that incubation with control AChE-huFc fused protein resulted in no noticeable binding (LDR-Fc control). Quantitation of the MFI in each scanned field (5 fields/lung) demonstrated a 70% increase in RBD binding in the lungs of LDR mice (Figure 7D). Whether this increased binding of RBD to LDR lungs is clinically functional and relevant needs to be further examined.

## Discussion

In the present study, we showed that transient pulmonary insults prompted by administration of low doses of the ALI/ARDS stimulants — LPS, bleomycin, or ricin — rendered mice sensitive to SARS-CoV-2 viral infection. Recently, mouse models were adapted for SARS-CoV-2 infection either by adjusting the viral spike RBD to fit the murine version of ACE2 (20, 21) or by transient or constitutive expression of human ACE2 in mice (7, 8). In contrast, in the model presented in the current study, mice expressed their native ACE2 receptor, yet were sensitive to genetically unaltered bona fide SARS-CoV-2. To this end, we employed an outbred animal model, CD-1 mice, for inducing ALI to better represent the variance in natural populations. CD-1 mice have been previously shown to display a robust and consistent ALI in response to LPS and ricin application (15, 22).

When LPS-treated mice were subjected to SARS-CoV-2 infection, the deleterious effect of the virus, reflected by additional loss of body weight, was slight, and the mice displayed body weight regain within a few days. This narrow dynamic range of measurable, virally induced body weight loss precludes the use of LPS-pretreated mice for meaningful evaluation of virus-induced pathologies or antiviral medical countermeasures. On the other hand, after SARS-CoV-2 infection of ricin- or bleomycin-pretreated mice, body weights of the mice decreased steadily and significantly during the entire course of the experimental surveillance period (15 days). Most importantly, 50%–100% of the ricin- and bleomycin-pretreated mice died after viral exposure.

The finding that SARS-CoV-2 sensitivity was retained even when the LDR mice were treated with anti-ricin antibodies before viral infection implies that active ricin was not required for conferring sensitivity toward the virus. Rather, the LDR-induced pulmonary pathologies themselves promoted viral susceptibility in these mice. Pathophysiological assessment of LDR mice at the time of viral infection identified various factors that may contribute to the pathological setting required for acquisition of sensitivity toward SARS-CoV-2. These include high-level expression of proinflammatory cytokines and chemokines in the lungs and serum, mild immune cell infiltration of the lungs, and elevated protein levels in the lungs, the latter indicative of compromised alveolar-capillary barrier function. Factors that may bring about gain of sensitivity toward SARS-CoV-2 in bleomycin-treated mice still need to be determined. It should be added in this context that the acquired sensitivity to SARS-CoV-2 may be related, at least in part, to an impairment of the immune response toward the virus as a result of the ALI stimulant pretreatment. Although such a possibility cannot be ruled out, we believe that reduced antiviral immune responses play at most a modest role in mouse sensitization to viral infection. This is because differences in viral infection were observed between treated and nontreated mice 2 days after infection, much earlier than the time required for T cell–related immune responses to occur. Moreover, if differential immune responses were to play a significant role in the disparate infectability of the 2 groups of mice observed at 2 days after infection, one would expect to observe a shift in the CD4^+^/CD8^+^ cell ratios of the group that mounted an immune response. However, measuring the CD4^+^/CD8^+^ cell ratios in the lungs and spleens of LDR-administered mice and sham-treated mice failed to establish any significant difference between the CD4^+^/CD8^+^ cell ratios in the 2 groups at the time of viral infection.

Confocal microscopy analysis demonstrated that LDR pretreatment led to increased SARS-CoV-2 binding to mouse lungs. In line with this observation, quantitative RT-PCR analysis of lungs derived from LDR–SARS-CoV-2 and SARS-CoV-2 mice demonstrated the presence of significantly higher levels of viral RNA in the lungs of LDR–SARS-CoV-2 mice, while growth kinetics and sgmRNA analyses in lung extracts testified to the presence of infectible and multiplying virus particles specifically in the lungs of LDR–SARS-CoV-2 mice. Viral growth kinetics and sgmRNA analyses demonstrated the presence of viable viral particles in lung extracts derived from bleomycin-pretreated mice as well. This finding is of special importance since it suggests that acquired susceptibility to SARS-CoV-2 is effectuated by ALI stimulant–induced lung injury as such.

High levels of viral RNA were also detected in the hearts and serum of LDR–SARS-CoV-2 mice and remained high at all examined time points. Conversely, viral RNA levels in the hearts and serum of SARS-CoV-2 mice were considerably lower and waned rapidly. A recently published study also reported the presence of extrapulmonary SARS-CoV-2 in organs such as the heart in a BALB/c-based mouse model infected by murine-adapted SARS-CoV-2 (21). This finding is of considerable clinical relevance because autopsy samples obtained from COVID-19 patients revealed the presence of the virus in organs other than the lungs, including the heart, brain, and blood (23). In some cases, brain and heart damage were also documented in COVID-19 patients (24), yet whether these injuries stem from direct viral activity or are due to an excessive immune response and virus-induced cytokine storm remains to be elucidated. Studies at our laboratory are now being performed to determine the presence and extent of organ injury in the hearts and brains of LDR–SARS-CoV-2 mice.

Taken together, our findings raise the intriguing question of how the virus gains entrance into cells of CD-1 mice. Protection of LDR–SARS-CoV-2 mice by application of anti-RBD antibodies seems to imply that viral entry into cells of LDR-treated mice is mediated via the RBD region of the viral spike. One may therefore be tempted to suggest that increased expression of ACE2 can compensate for the relatively poor binding of SARS-CoV-2 spike RBD to this ACE2 species and that overexpression of this receptor in mouse lung cells can overcome this limitation and cause a successful viral infection. However, comparative flow cytometry analysis of lung cells derived from sham-treated and LDR mice failed to detect an increase in ACE2-expressing cells after application of LDR. On the other hand, histochemical analysis of lung sections prepared from LDR mice did reveal a marked increase in the binding of viral RBD. The combination of these 2 findings may imply that in LDR mice (and possibly in bleomycin mice), there is a gain of receptors other than ACE2 that can serve as an effective binding site for SARS-CoV-2, perhaps with the aid of the high level of lung cell surface TMPRSS2 induced by LDR application. It is interesting to note in this context that TMPRSS2 was implied to promote SARS-CoV entry not only by proteolytic modification of the viral spike protein but also by modifying the ACE2 receptor itself (25). It may be that the excess TMPRSS2 observed in pulmonary epithelial cells of LDR mice plays a role in the generation of new target sites for SARS-CoV-2 binding and entry by proteolytic modification of existing membrane surface proteins.

Various viruses use junction proteins as receptors and access epithelium cells through apical junction complexes that contain tight junctions and adherens junctions, even if most of these proteins are not readily available, possibly by taking advantage of compromised epithelium (26). Indeed, studies carried out in our laboratory demonstrated that i.n. application of a lethal dose of ricin (7 μg/kg) to mice leads to rapid diminution of adherens and tight junctions in the lungs, thereby precipitating the disruption of the alveolar-capillary barrier (27). Intriguingly, altered expression of tight junction molecules in the alveolar septa has also been reported in bleomycin- and LPS-induced lung injury models (28, 29). In line with the notion that alterations in junction protein integrity play a role in the acquired sensitivity to SARS-CoV-2, GO analysis of the RNA-Seq upregulated genes in lungs of LDR mice at the time of viral infection demonstrated the enrichment of pathways involved in protein binding, including enrichment of gene clusters involved in the cadherin binding pathway (molecular function in Supplemental Table 2).

Whether aberrations of junction proteins indeed play a significant role in the sensitization of mice toward SARS-CoV-2 remains to be determined. Nevertheless, and irrespective of the exact mechanism by which SARS-CoV-2 gains entrance to CD-1 mouse lung cells, the existence of second tier entry points for SARS-CoV-2 in the case of preexisting tissue injury may be of relevance to the development of severe COVID-19 in high-risk populations that may have compromised tissue integrity. Decoding the process by which SARS-CoV-2 gains entrance into murine cells after sensitization by ricin or bleomycin is therefore a focal point of the ongoing research at our laboratory.

In summary, we established a mouse model for the study of the molecular pathways involved in SARS-CoV-2–induced pathologies over tissue injury background and for examining medical countermeasures that may be beneficial for treatment of COVID-19 in high-risk populations.

## Methods

### Cells and virus.

African green monkey kidney clone E6 cells (Vero E6, ATCC, CRL-1586) were grown in DMEM containing 10% FBS, MEM nonessential amino acids, 2 mM l-glutamine, 100 units/mL penicillin, 0.1 mg/mL streptomycin, and 12.5 units/mL nystatin (Biological Industries). Cells were cultured at 37°C, 5% CO_2_ at 95% air atmosphere.

SARS-CoV-2 (GISAID accession EPI_ISL_406862) was provided by Bundeswehr Institute of Microbiology, Munich, Germany. Virus stocks were propagated (4 passages) and titered on Vero E6 cells. SARS-CoV-2 studies were conducted in a biosafety level 3 facility in accordance with the biosafety guidelines of the Israel Institute for Biological Research.

### Ricin, bleomycin, and LPS.

Crude ricin was prepared by us from seeds of endemic *Ricinus communis*, essentially as described before (13). Bleomycin sulfate (from *Streptomyces verticillus*, B8416-15U) and LPS (from *Escherichia coli*, L4391) were purchased from MilliporeSigma.

### Animal experiments.

Animals in this study were female CD-1 mice (Charles River Laboratories) weighing 27–32 g. Prior to treatment or infection, animals were habituated to the experimental animal unit for at least 5 days. All mice were housed in filter-top cages in an environmentally controlled room and maintained at 21°C ± 2°C and 55°C ± 10% humidity. Lighting was set to mimic a 12-hour light/12-hour dark cycle. Animals had access to food and water ad libitum. Treatment of animals was in accordance with regulations outlined in the US Department of Agriculture Animal Welfare Act and the conditions specified in the NIH *Guide for Care and Use of Laboratory Animals* (National Academies Press, 2011).

Ricin (1.7 μg/kg), bleomycin (2 U/kg), or LPS (1.7 mg/kg) was i.n. applied (25 μL per nostril) to mice anesthetized by an i.p. injection of ketamine (1.9 mg/mouse) and xylazine (0.19 mg/mouse). Mice displaying weight loss of more than 30% were euthanized by cervical dislocation.

SARS-CoV-2 diluted in PBS supplemented with 2% FBS was i.n. instilled to mice anesthetized as above. Where indicated, mice were administered (i.p. injection, 1 mL) one of the following antibodies: equine-derived polyclonal anti-ricin F(ab)_2_ fragment (1730 IsNU) (14), monoclonal anti–SARS-CoV-2 RBD (MD65) antibody (1 mg) (18), polyclonal anti–SARS-CoV-2 RBD antibody (SARS-CoV-2 NT_50_ = 1:20,000), or polyclonal anti–SARS-CoV-2 (SARS-CoV-2 NT_50_ = 1:10,000) antibody. Polyclonal anti–SARS-CoV-2 RBD antibody was generated by immunizing a rabbit (female New Zealand white) with RBD-huFc (150 μg, complete Freund’s adjuvant) followed by boosting with RBD-huFc (150 μg, incomplete Freund’s adjuvant) at day 21 and with monomeric RBD (150 μg, incomplete Freund’s adjuvant) at day 42. Hyperimmune serum was collected at day 50. RBD-huFc-and monomeric RBD were expressed and purified as described (18). Polyclonal anti–SARS-CoV-2 antibody was generated in a rabbit (female New Zealand white) by repeated i.v. injection of live SARS-CoV-2 (1 × 10^6^ PFU at days 0, 7, 10, 14, and 17). Serum was collected 14 days after the last virus injection.

### Mouse activity measurement.

Nocturnal activity of mouse groups was monitored utilizing a computerized home cage monitoring system (HCMS100) with a single laser beam and detector as described previously (13).

### Clinical laboratory analysis.

Differential blood counts were determined in peripheral blood. Samples were collected from the tail vein of mice into EDTA-containing tubes (BD Biosciences) and were analyzed using the Veterinary Multi-species Hematology System Hemavet 850 (Drew Scientific).

### BALF analysis.

BALF collected by instillation of 1 mL PBS at room temperature via a tracheal cannula was centrifuged at 240*g* at 4°C for 10 minutes. Supernatants were collected and stored at –20°C until further use. BALF levels of IL-6, IL-1β, and TNF-α were determined using ELISA kits following the manufacturer’s instructions (R&D Systems, Bio-Techne). Protein concentrations in BALF samples were determined by a NanoDrop 2000 spectrophotometer (Thermo Fisher Scientific). Cholinesterase levels were determined as described before (15).

### Measurement of ricin catalytic activity.

Ricin-induced depurinated 28S rRNA in mouse lungs was measured as described previously (30) and expressed as a percentage of total 28S rRNA.

### Measurement of viral RNA.

Mice lungs, trachea, nasal turbinate, heart, spleen, liver, kidney, and brain were harvested and ground. Serum was separated from collected blood. From each sample, 200 μL of clarified lysate or serum was added to lysis buffer (supplied with the kit) and viral RNA was extracted using the RNAdvance viral kit (Beckman Coulter) and further processed on the Biomek i7 Automated Workstation (Beckman Coulter) according to the manufacturer’s protocol. Each sample was eluted in 50 μL of RNase-free water. Real-time RT-PCR assays targeting the SARS-CoV-2 E gene were performed using the SensiFAST Probe Lo-ROX One-Step kit (Bioline). The final concentration of primers was 600 nM and probe concentration was 300 nM. Primers and probe for the E gene assay were taken from the Berlin protocol published in the WHO recommendation for the detection of SARS-CoV-2 (31). The primers and probe sequences were as follows: E_Sarbeco_F1 ACAGGTACGTTAATAGTTAATAGCGT, E_Sarbeco_R2 ATATTGCAGCAGTACGCACACA, E_Sarbeco_P1 ACACTAGCCATCCTTACTGCGCTTCG.

Thermal cycling was performed at 48°C for 20 minutes for reverse transcription followed by 95°C for 2 minutes, 45 cycles of 94°C for 15 seconds, and 60°C for 35 seconds. Ct values were converted to calculated PFUs with the aid of a calibration curve tested in parallel.

RT-PCR assays targeting the sgmRNA of the E gene were performed essentially as described (32) using the forward primer sgLeadSARSCoV2-F CGATCTCTTGTAGATCTGTTCTC and the reverse primer and probe E_Sarbeco_R2 and E_Sarbeco_P1 as described above. Thermal cycling was 48°C for 20 minutes for reverse transcription followed by 95°C for 3 minutes, 45 cycles of 95°C for 10 seconds, 56°C for 15 seconds, and 72°C for 5 seconds. Ct values were converted to calculated PFUs with the aid of a calibration curve tested in parallel.

### Viral growth kinetics.

Viral growth kinetics were measured essentially as previously described (33). Briefly, lung homogenates (1:10 in DMEM) were incubated with Vero E6 cells in 24-well culture plates for 40 minutes (37°C 5% CO_2_); cells were washed 4 times with PBS and 1 time with DMEM and then incubated with DMEM (see above). Supernatant samples (200 μL) removed at 0, 24, and 48 hours were added to LBF lysis buffer (see above) and subjected to RT-PCR as described above.

### RNA-Seq.

RNA was isolated from lungs of mice using RNeasy Mini Kits with an on-column DNase step according to the manufacturer’s instructions (QIAGEN). RNA quantification was carried out in a Qubit fluorometer using the Qubit RNA HS assay kit (Invitrogen, Thermo Fisher Scientific).

RNA-Seq was performed at the JP Sulzberger Columbia Genome Center (New York, New York, USA). Libraries were generated using the Illumina TruSeq Standard mRNA kit according to the manufacturer’s instructions. Polyadenylated RNA enrichment was performed. Sequencing of 100 bp paired-end reads was performed on the Illumina NovaSeq 6000 system. RNA-Seq quality control was performed using fastQC v0.11.5, checking for per-base sequence quality, per-sequence quality scores, and adapter content. Pseudoalignment to a Kallisto index created from transcriptomes (Mouse: GRCm38) was performed using Kallisto (0.44.0). We verified that each sample reached at least 75% of the target read goal and checked for adequate sequence alignment percentages. Sequencing yielded 19.5 M to 25.1 M reads per sample, resulting in identification of 35,199 transcripts. Analysis of DEGs under various conditions was performed using the R package DESeq2 v1/18.1 with default parameters. The mouse GRCm38 annotation file was downloaded from the ensemble BioMart website (https://www.ensembl.org/Mus_musculus/Info/Index). GO enrichment analysis was carried out with Fisher’s exact test to estimate the specific GO categories using OmicsBox software (https://www.biobam.com/omicsbox).

### Histology.

Lungs were rapidly isolated, carefully inflated, and fixed in 4% natural-buffered paraformaldehyde at room temperature for 2 weeks followed by routine processing for paraffin embedding. Serial sections 5 μm thick were cut, and selected sections were stained with H&E for general histopathology and with Masson’s trichrome for collagen and examined by light microscopy. Images were acquired using the Panoramic MIDI II slide scanner (3DHISTEC).

### Confocal microscopy.

Lungs were processed as above and sections (5 μm) were mounted on glass slides and deparaffinized. Antigen retrieval was performed by incubation in target retrieval solution (DAKO, 30 minutes, 95°C). After blocking in 5% BSA in PBS, slides were incubated (overnight, 4°C) with SARS-CoV-2 (1000 PFU/mL) or with RBD-huFc fused protein (18), and detection was performed using anti–SARS-CoV-2 spike mAb (generated as described in ref. 18 for anti–SARS-CoV-2 RBD mAb) followed by Alexa Fluor 594–labeled anti-human antibody for virus binding detection (Molecular Probes, Thermo Fisher Scientific) or anti-human Alexa Fluor 594 antibody for RBD-huFc binding detection. AChE-huFc fused protein served as a negative control (19) in RBD binding experiments. For nuclear staining, slides were mounted with ProLong Gold Antifade Mountant containing DAPI (Molecular Probes, Thermo Fisher Scientific). Analysis was performed using an LSM 710 confocal scanning microscope (Zeiss) equipped with the following lasers: argon multiline (458/488/514 nm), diode 405 nm, diode-pumped solid-state 561 nm, and helium-neon 633 nm. Fluorescence intensity was quantified using Zen software (version 2.1, Zeiss).

### Flow cytometry.

Lungs were harvested, cut into small pieces, and digested (1 hour, 37°C) with 4 mg/mL collagenase D (Roche). Tissue was meshed through a 40 μm cell strainer, and RBCs were lysed. Cells were stained using the following antibodies (eBioscience, Thermo Fisher Scientific): CD45-FITC (clone 30-F11), CD31-PE (clone 390), and CD326-PerCP (clone G8.8). Cells were defined as endothelial cells (CD45^–^CD31^+^) and epithelial cells (CD45^–^CD31^–^CD362^+^). For ACE2 receptor staining, cells were stained using goat anti-mACE2 antibody (R&D Systems, Bio-Techne, AF3437) followed by donkey anti-goat IgG coupled to AF647 (Thermo Fisher Scientific, A20186). For TMPRSS2 staining, cells were stained using anti-TMPRSS2 polyclonal antibody coupled to AF647 (Santa Cruz Biotechnology, sc-515727). Cells were analyzed on FACSCalibur (BD Biosciences).

### Data and materials availability.

The transcriptomic data have been deposited in NCBI’s Gene Expression Omnibus (GEO) (accession number of the transcriptome series is GSE159461 and the SRA BioProject number is PRJNA669006).

### Statistics.

Statistical analysis was performed using Prism software (version 5.01, 2007; GraphPad). All data are presented as means ± SEM. Simple comparisons were performed using the unpaired 2-tailed Student’s *t* test. A 2-way ANOVA test was performed for multiple comparisons followed by a Bonferroni posttest. Significance was set at *P* less than 0.05.

### Study approval.

Animal experiments were performed in accordance with Israeli law and were approved by the Ethics Committee for Animal Experiments at the Israel Institute for Biological Research (project identification codes M-23-20, M-25-20, M-44-20, M-46-20, M-49-20).

## Author contributions

RF, CK, TS, and SY conceived and designed the experiments. RF, LBO, SL, TK, HG, MA, DG, YE, EM, ICG, GZ, OS, TA, OI, DS, OM, EZ, YV, SM, BP, HA, CK, and TS conducted the investigation. HL, OM, RR, RA, TI, SM, HA, IG, and ABS provided resources. CK and TS wrote the original draft. RF, OM, LBO, SL, TK, TI, SM, BP, HA, ICG, and RR reviewed the manuscript.

## Supplementary Material

Supplemental data

## Version 1 05/11/2021

In-Press Preview

## Version 2 06/22/2021

Electronic publication
