# Supplementary material for: Mice with induced pulmonary morbidities display severe lung inflammation and mortality following exposure to SARS-CoV-2
Source: JCI Insight. 2021 Jun 22;6(12):e145916. doi: 10.1172/jci.insight.145916 (PMC8262502; doi:10.1172/jci.insight.145916)
Supplement: Supplemental data [file jciinsight-6-145916-s032.pdf]

Supplemental Table 1: Blood counts, BALF analyses and ricin catalysis products in CD-1 mice following application of low-dose ricin

		**sham mice**	**LDR mice-day 0** (*p* value *vs*. sham)	**LDR mice-day 7** (*p* value *vs*. sham)
**complete blood count**[A]	WBC (K/μl)	4.51±1.68	10.64±1.78 (**0.003**)	6.04±4.72 (0.564)
	NEU (K/μl)	0.58± 0.92	1.82±4.81 (**0.007**)	1.39±1.18 (0.496)
	MO (K/μl)	0.17±0.17	0.73±0.22 (**0.007**)	0.14±0.14 (0.809)
	PLT (K/μl)	499±220	942±197 (**0.024**)	734±217 (0.205)
**bronchoalveolar lavage fluid**[B]	protein (mg/ml)	1.56±0.21	2.01±0.18 (**0.006**)	4.44±1.7 (**0.007**)
	cholinesterase (mOD/min)	<5	355±143 (**0.0001**)	<5
	cells ($x10^6$)	36±27	86±27 (**0.019**)	17.2±7.6 (0.178)
	IL-6 (pg/ml)	34±29	586±110 (**4.47E-06**)	75±30 (0.06)
	IL-1β (pg/ml)	1±2	28±15 (**0.004**)	43±41 (0.052)
	TNF-α (ng/ml)	8±12	38±12 (**0.005**)	28±22 (0.109)
**ricin catalysis products in lungs**[B]	depurinated 28S rRNA (%)	0.22±0.31	7.94±3.11 (**0.00056**)	0.42±0.6 (0.53)

[A] n=4; [B] n=5. Statistically significant *t*-test values (p<0.05) are presented in bold type.

Supplemental Table 2: Significant upregulated GO terms*

Biological Process:

GO ACCESSION	GO TERM	FDR	# of DEGs
GO:0050911	detection of chemical stimulus involved in sensory perception of smell	1.49E-29	7
GO:0070098	chemokine-mediated signaling pathway	2.70E-16	33
GO:0035458	cellular response to interferon-beta	3.03E-12	17
GO:0010469	regulation of signaling receptor activity	1.41E-11	99
GO:0051092	positive regulation of NF-kappaB transcription factor activity	5.39E-11	35
GO:0045071	negative regulation of viral genome replication	1.94E-10	21
GO:0032755	positive regulation of interleukin-6 production	4.51E-10	27
GO:0042832	defense response to protozoan	7.56E-10	17
GO:0010575	positive regulation of vascular endothelial growth factor production	1.05E-09	14
GO:0006270	DNA replication initiation	4.35E-09	16
GO:0032729	positive regulation of interferon-gamma production	7.02E-09	25
GO:0050830	defense response to Gram-positive bacterium	9.95E-09	28
GO:0043123	positive regulation of I-kappaB kinase/NF-kappaB signaling	7.80E-08	34
GO:0032735	positive regulation of interleukin-12 production	2.86E-07	14
GO:0000028	ribosomal small subunit assembly	6.65E-07	10
GO:0000281	mitotic cytokinesis	8.91E-07	17
GO:1990830	cellular response to leukemia inhibitory factor	1.51E-06	32
GO:0001916	positive regulation of T cell mediated cytotoxicity	1.94E-06	10
GO:0032715	negative regulation of interleukin-6 production	2.23E-06	16
GO:0048535	lymph node development	2.71E-06	13
GO:1904874	positive regulation of telomerase RNA localization to Cajal body	4.33E-06	9
GO:0032689	negative regulation of interferon-gamma production	5.66E-06	14
GO:0000463	maturation of LSU-rRNA from tricistronic rRNA transcript (SSU-rRNA, 5.8S rRNA, LSU-rRNA)	8.92E-06	8
GO:0070431	nucleotide-binding oligomerization domain containing 2 signaling pathway	8.92E-06	8
GO:0045651	positive regulation of macrophage differentiation	8.92E-06	8
GO:0090023	positive regulation of neutrophil chemotaxis	9.49E-06	11
GO:0048147	negative regulation of fibroblast proliferation	1.46E-05	13
GO:1904871	positive regulation of protein localization to Cajal body	2.03E-05	6
GO:0042531	positive regulation of tyrosine phosphorylation of STAT protein	2.67E-05	16
GO:1903955	positive regulation of protein targeting to mitochondrion	3.46E-05	21
GO:0032733	positive regulation of interleukin-10 production	3.75E-05	12
GO:0032731	positive regulation of interleukin-1 beta production	3.75E-05	12
GO:0045730	respiratory burst	5.79E-05	11
GO:0032743	positive regulation of interleukin-2 production	6.06E-05	12
GO:0043011	myeloid dendritic cell differentiation	6.07E-05	9
GO:1901998	toxin transport	6.39E-05	15
GO:0090026	positive regulation of monocyte chemotaxis	6.90E-05	8
GO:0046641	positive regulation of alpha-beta T cell proliferation	8.54E-05	10
GO:0051897	positive regulation of protein kinase B signaling	9.12E-05	21
GO:0038094	Fc-gamma receptor signaling pathway	1.14E-04	6
GO:0071359	cellular response to dsRNA	1.20E-04	9
GO:0032211	negative regulation of telomere maintenance via telomerase	1.20E-04	9
GO:1900017	positive regulation of cytokine production involved in inflammatory response	1.20E-04	9
GO:0043547	positive regulation of GTPase activity	1.26E-04	57
GO:0043124	negative regulation of I-kappaB kinase/NF-kappaB signaling	1.29E-04	13
GO:0002925	positive regulation of humoral immune response mediated by circulating immunoglobulin	1.64E-04	5
GO:0071224	cellular response to peptidoglycan	1.64E-04	5
GO:2001268	negative regulation of cysteine-type endopeptidase activity involved in apoptotic signaling pathway	1.64E-04	5
GO:0075525	viral translational termination-reinitiation	1.64E-04	5
GO:0032728	positive regulation of interferon-beta production	2.18E-04	12

Molecular Function:

GO ACCESSION	GO TERM	FDR	# of DEGs
GO:0003735	structural constituent of ribosome	2.79E-59	97
GO:0004984	olfactory receptor activity	1.49E-29	7
GO:0005524	ATP binding	1.78E-27	266
GO:0008009	chemokine activity	9.22E-16	25
GO:0005525	GTP binding	7.81E-11	77
GO:0003743	translation initiation factor activity	3.54E-10	23
GO:0045296	cadherin binding	4.29E-10	59
GO:0003924	GTPase activity	9.02E-10	60
GO:0031625	ubiquitin protein ligase binding	2.08E-09	58
GO:0008270	zinc ion binding	2.66E-09	115
GO:0042803	protein homodimerization activity	5.68E-09	117
GO:0004298	threonine-type endopeptidase activity	2.23E-08	13
GO:0003697	single-stranded DNA binding	2.44E-08	27
GO:0005164	tumor necrosis factor receptor binding	2.86E-07	14
GO:0003725	double-stranded RNA binding	3.05E-07	23
GO:0048027	mRNA 5'-UTR binding	1.94E-06	10
GO:0046982	protein heterodimerization activity	2.86E-06	75
GO:0017116	single-stranded DNA helicase activity	4.33E-06	9
GO:0051059	NF-kappaB binding	4.62E-06	11
GO:0005031	tumor necrosis factor-activated receptor activity	9.49E-06	11
GO:0000978	RNA polymerase II cis-regulatory region sequence-specific DNA binding	1.18E-05	78
GO:0001530	lipopolysaccharide binding	1.83E-05	11
GO:0047485	protein N-terminus binding	1.93E-05	23
GO:0036402	proteasome-activating ATPase activity	2.03E-05	6
GO:0000049	tRNA binding	2.11E-05	15
GO:0001054	RNA polymerase I activity	2.73E-05	8
GO:0003730	mRNA 3'-UTR binding	5.80E-05	17
GO:0001056	RNA polymerase III activity	6.07E-05	9
GO:0003746	translation elongation factor activity	6.07E-05	9
GO:0008017	microtubule binding	1.08E-04	46
GO:0016176	superoxide-generating NADPH oxidase activator activity	1.14E-04	6
GO:0001228	DNA-binding transcription activator activity, RNA polymerase II-specific	1.47E-04	65
GO:1990825	sequence-specific mRNA binding	1.64E-04	5
GO:0097677	STAT family protein binding	1.64E-04	5
GO:0036002	pre-mRNA binding	2.46E-04	10
GO:0051082	unfolded protein binding	3.35E-04	19
GO:0043130	ubiquitin binding	4.05E-04	16
GO:0003755	peptidyl-prolyl cis-trans isomerase activity	4.55E-04	12
GO:0043022	ribosome binding	5.39E-04	14
GO:0042056	chemoattractant activity	6.07E-04	10
GO:0051117	ATPase binding	6.65E-04	16
GO:0003724	RNA helicase activity	6.65E-04	16
GO:0003713	transcription coactivator activity	7.32E-04	40
GO:0061631	ubiquitin conjugating enzyme activity	7.77E-04	11
GO:0010997	anaphase-promoting complex binding	7.77E-04	5
GO:0043023	ribosomal large subunit binding	7.77E-04	5
GO:0051539	4 iron, 4 sulfur cluster binding	8.73E-04	12
GO:0008137	NADH dehydrogenase (ubiquinone) activity	9.63E-04	8
GO:0004550	nucleoside diphosphate kinase activity	9.63E-04	8
GO:0003887	DNA-directed DNA polymerase activity	9.74E-04	9

***** Top 50 GO terms of biological processes and molecular function terms, with their false discovery rate (FDR) value and the number DEGs in lungs of LDR mice compared to naïve (n=4).

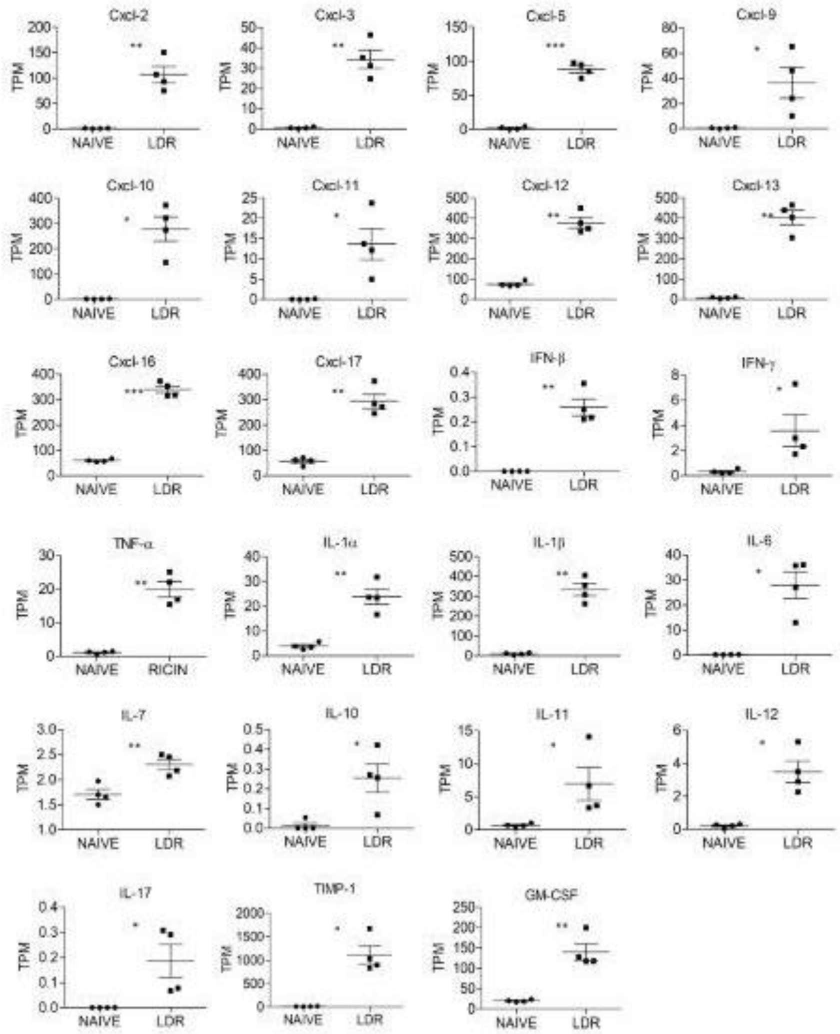

Supplemental Figure 1: Effects of low dose ricin application on cytokine and chemokine gene expression in lungs of CD-1 mice. Normalized gene expressed levels (transcripts per million, TPM) of 23 indicated cytokines and chemokines determined from RNAseq data of LDR mice (2 days post-low-dose-ricin-application) compared with naïve control. Data are represented as average $\pm$ SEM (n=4 per group) analyzed using unpaired t-test. *p <0.05, **p <0.01, ***p <0.001

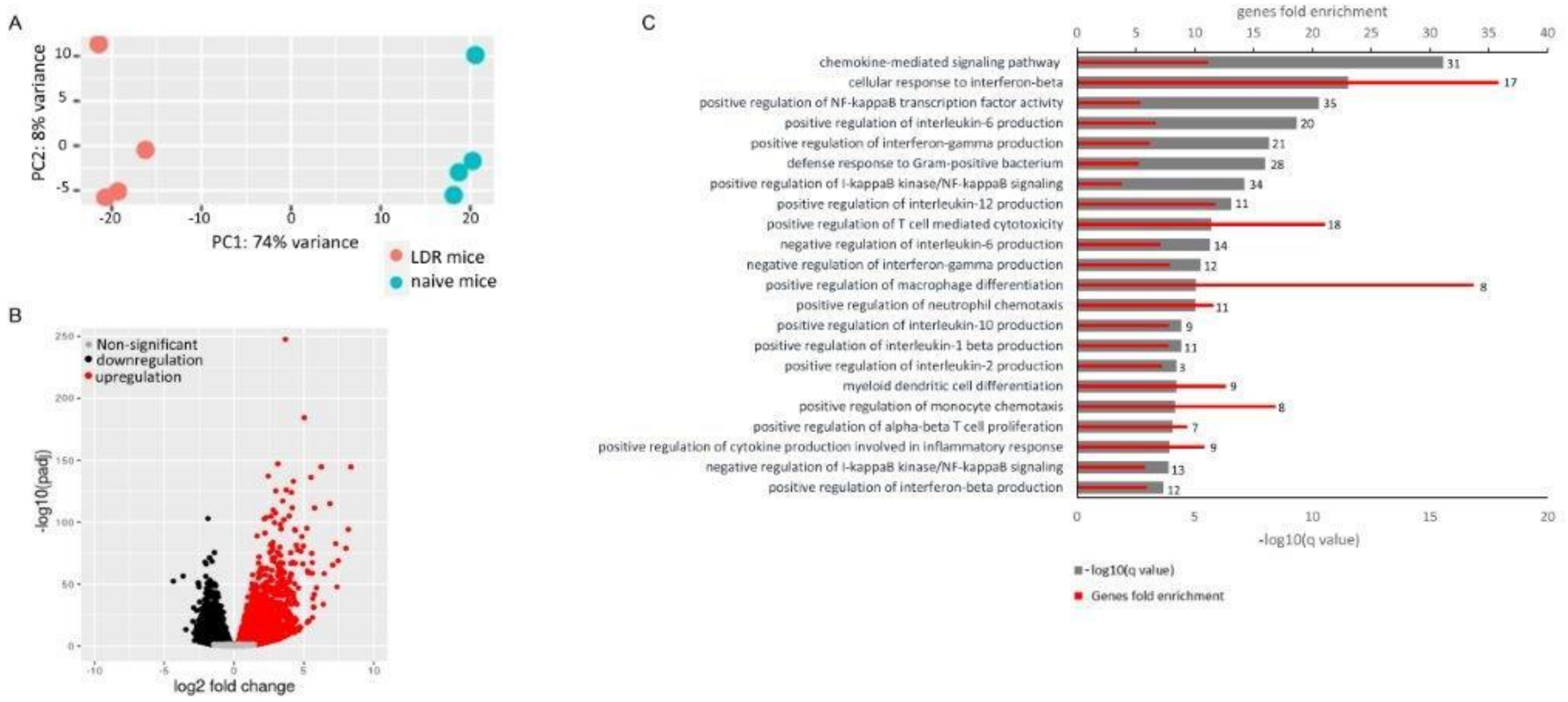

Supplemental Figure 2: Distinct transcriptional signatures in LDR mice compared to naïve mice. RNAseq analysis of lung homogenates from naïve- and LDR- (2 days post-low-dose-ricin application) mice (n=4). A: Principal component analysis was performed for 8 samples with log2 transformed gene-level normalized counts data. PC, principal component. B: Volcano plot comparing differentially expressed genes, respectively, with a false discovery rate (q value) < 0.05. Multiple comparisons were accounted for by calculation of a Benjamini-Hochberg false discovery rate-adjusted P value (q value). Each dot in the volcano plot represents a single gene. C: Gene Ontology (GO) enrichment analysis of biological process (BP) terms enriched in upregulated genes from comparisons of LDR mice versus naïve. Terms were ranked by the false discovery rate (q value) and the immune and inflammatory terms are listed from the top 50 after eliminating redundant terms. Gray bars show the –log10[q value]. Red bars show the genes fold enrichment.

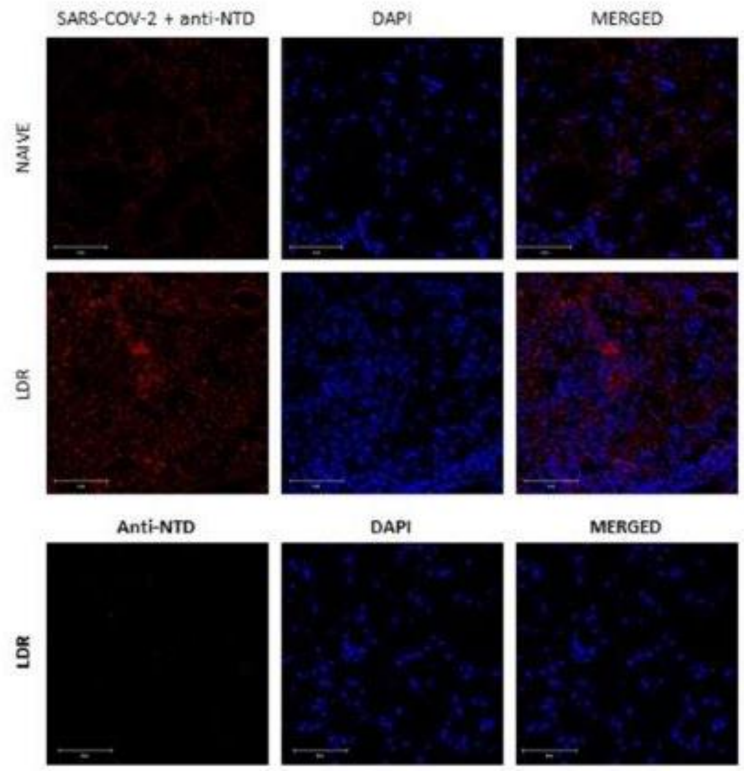

Supplemental Figure 3: Confocal microscopy scans of lung sections. Lung sections prepared from naïve (upper row) or LDR mice at 2 days post-ricin administration (middle row) were incubated with SARS-CoV-2 (1000 PFU/ml) and then immunostained with a monoclonal antibody directed against the spike region of the SARS-CoV-2, followed by AF594-labeled anti-human antibody. Lung sections of LSR mice incubated with vehicle only (no virus) served as a negative control for the anti-SARS-CoV-2 monoclonal antibody (bottom row). Staining of SARS-CoV-2 in red and identification of nuclei by DAPI in blue, bar= 50 µm. Representative sections of n=4 mice are shown.

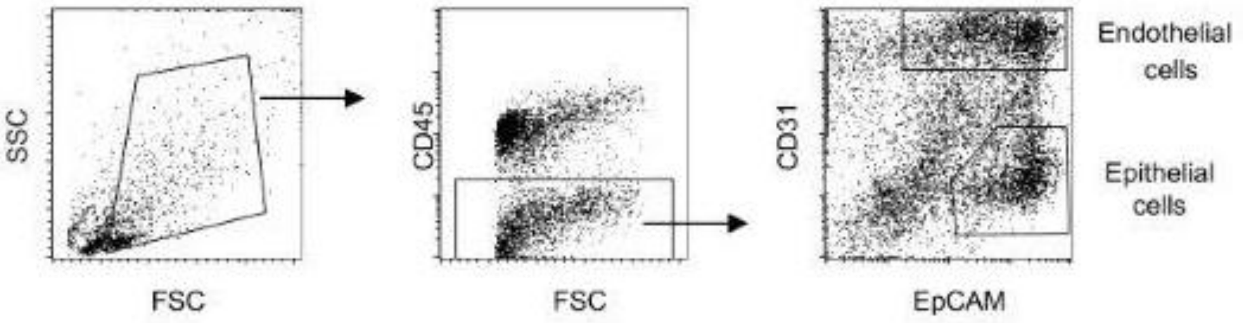

Supplemental Figure 4: A hierarchical gating strategy to identify lung epithelial and endothelial cells. Representative FACS plot (n=4) that identifies lung epithelial cells (CD45-CD31-EpCAM+) and endothelial cells (CD45-CD31+)
